# The role of chest imaging in the diagnosis, management, and monitoring of coronavirus disease 2019 (COVID-19)

**DOI:** 10.1186/s13244-021-01096-1

**Published:** 2021-11-02

**Authors:** Shohei Inui, Wataru Gonoi, Ryo Kurokawa, Yudai Nakai, Yusuke Watanabe, Keita Sakurai, Masanori Ishida, Akira Fujikawa, Osamu Abe

**Affiliations:** 1 Department of Radiology, Graduate School of Medicine, The University of Tokyo, 7-3-1, Hongo, Bunkyo-ku, Tokyo, 113-8655 Japan; 2 Department of Radiology, Japan Self-Defense Forces Central Hospital, 1-2-24, Ikejiri, Setagaya-ku, Tokyo, 154-0001 Japan; 3 Division of Neuroradiology, Department of Radiology, University of Michigan, 1500 E Medical Center Dr, UH B2, Ann Arbor, MI 48109 USA; 4 Department of Radiology, National Center for Geriatrics and Gerontology, 7-430, Morioka-cho, Obu, Aichi 474-8511 Japan

**Keywords:** SARS-CoV-2, 2019 novel coronavirus, COVID-19, CO-RADS, The RSNA expert consensus statement

## Abstract

Coronavirus disease 2019 (COVID-19) pandemic has posed a major public health crisis all over the world. The role of chest imaging, especially computed tomography (CT), has evolved during the pandemic paralleling the accumulation of scientific evidence. In the early stage of the pandemic, the performance of chest imaging for COVID-19 has widely been debated especially in the context of comparison to real-time reverse transcription polymerase chain reaction. Current evidence is against the use of chest imaging for routine screening of COVID-19 contrary to the initial expectations. It still has an integral role to play, however, in its work up and staging, especially when assessing complications or disease progression. Chest CT is gold standard imaging modality for COVID-19 pneumonia; in some situations, chest X-ray or ultrasound may be an effective alternative. The most important role of radiologists in this context is to be able to identify those patients at greatest risk of imminent clinical decompensation by learning to stratify cases of COVID-19 on the basis of radiologic imaging in the most efficient and timely fashion possible. The present availability of multiple and more refined CT grading systems and classification is now making this task easier and thereby contributing to the recent improvements achieved in COVID-19 treatment and outcomes. In this article, evidence of chest imaging regarding diagnosis, management and monitoring of COVID-19 will be chronologically reviewed.

## Key points

- Chest CT is gold standard imaging modality for COVID-19 pneumonia; in some situations, chest X-ray or ultrasound may be an effective alternative.
- Chest imaging is not recommended for routine screening of COVID-19 in a resource-abundant environment.
- Chest imaging is useful for assessing complications, disease progression, and prognostication of COVID-19.
- Proposed chest CT classification systems are useful for stratifying cases suspected of COVID-19.

## Introduction

The outbreak of coronavirus disease 2019 (COVID-19) has begun in Wuhan, Hubei province, China, and rapidly spread all over the world [1]. The World Health Organization (WHO) declared it a Public Health Emergency of International Concern on January 30, 2020 and a pandemic on March 11, 2020 [1, 2]. This latter designation facilitated rigorous investigation and multinational large-scale study, with the rate of evidence accumulating explosively. Now this condition that was first reported as “pneumonia of unknown cause” has been profiled to a considerable degree [3].

The role of chest imaging, especially computed tomography (CT), has evolved during the pandemic paralleling the accumulation of scientific evidence. The performance of chest imaging for COVID-19 has widely been debated since the early period of the pandemic especially in the context of comparison to real-time reverse transcription polymerase chain reaction (RT-PCR). In this article, evidence of chest imaging regarding diagnosis, management and monitoring of COVID-19 will be chronologically reviewed.

## Controversy of chest CT versus RT-PCR

Patients with SARS-Cov-2 infection can experience a diverse range of clinical presentations, from no symptoms to acute respiratory distress syndrome (ARDS), septic shock, and/or multiple organ failure [4, 5]. SARS-Cov-2 virus is detectable in the respiratory tract 2–3 days before symptom onset, peaks at symptom onset, and declines over the following 7–8 days [6]. Difficulties in infection control of COVID-19 are in part ascribable to this viral shedding profile, contrasting to that of influenza virus that peaks after symptom onset [7]. RT-PCR is currently the most reliable diagnostic tool for COVID-19 [8]. Specimens obtained from a nasopharyngeal or oropharyngeal swab are commonly used [7]. However, the false-negative rate of RT-PCR test is not negligible, estimated as 100% on the day of infection (day1) and 38% on the day of symptom onset (day 5), which decreases to 20% at 3 days after symptom onset (day 8) and increases again thereafter [9]. The instability of RT-PCR may be ascribed to variabilities in viral load depending on the disease stage and sampling error [5, 7, 8]. Bronchoalveolar lavage is more sensitive than RT-PCR but not realistic for route application [10]. In addition, in the early phase of the pandemic, the use of RT-PCR was limited because of logistical issues—including the development, mass production, and dissemination of the examination kit. The turnaround time of RT-PCR was several days in early 2020 [11].

Given these limitations of RT-PCR, the use of chest CT was widely debated since the early period of the pandemic, especially in the context of replacing RT-PCR as a diagnostic tool [12, 13]. Chest CT was deemed more available in many hospitals and possibly able to achieve a superior diagnostic performance in the early period of infection [14]. Early radiological studies from China spurred this discussion. On February 2020, a study from China first reported that 5 symptomatic patients showed chest CT abnormalities despite initial PCR negative results [15]. A subsequent study from China reported that chest CT showed a higher sensitivity than RT-PCR (98% vs. 71%) [16]. Similar findings were also reported from a larger cohort study from China that investigated 1014 patients and revealed the sensitivity of chest CT and RT-PCR to be 97% and 88%, respectively, based on which they recommended chest CT for screening of COVID-19 instead of RT-PCR [17]. Finally, a meta-analysis from Korea summarizing early reports published within the first 1 month of the pandemic reported that the sensitivity of chest CT exceeds that of RT-PCR (93% vs. 89%) with a specificity of 37% [18].

However, problems were noted in many of the early studies published within 1 months of the pandemic: (1) Patient background was not specified; (2) disease severity of the cohort was biased toward severe and hospitalized cases; (3) the indications for performing chest CT scan were not described; (4) a definition of positive chest CT findings was not provided (despite positive CT findings were not equal to positive CT findings of COVID-19); (5) nonuniformity of the reference standard [19–22]. Antithesis of screening by chest CT was presented in March 2020 by a Japanese study of a mass infection cohort, reporting the sensitivity of chest CT to be 79% in symptomatic and 54% in asymptomatic patients [23, 24].

Based on a risk–benefit analysis including diagnostic performance, medical cost, precaution issue, and risk of radiation exposure, medical specialty societies published position statements against the use of chest CT for screening of COVID-19 including the American College of Radiology (ACR), the Society of Thoracic Radiology (STR), and the American Society of Emergency Radiology (ASER) in March to April, 2020 [12, 25, 26]. The Fleischner Society also published a consensus statement of a multidisciplinary expert panel [27]. Their statement offered guidance for the use of chest imaging modalities in different healthcare environments and scenarios [27]. The multidisciplinary panel concluded that chest CT is not recommended for asymptomatic or mild symptomatic patients with COVID-19 in the absence of accompanying risk factors or routine screening in a resource abundant environment [27]. On the other hand, they recommended chest CT for medical triage of patients with suspected COVID-19, who present with moderate to severe clinical features and a high-pretest probability of disease in a resource-constrained environment [27]. They also recommended chest CT for patients with moderate to severe symptoms with suspected COVID-19 or those experiencing respiratory functional impairment, hypoxemia, or both after recovery from infection [27]. For these patients, imaging provides a baseline for future comparison, may reveal an alternative diagnosis, may establish manifestations of important comorbidities in patients with risk factors for disease progression, and may influence treatment strategy and the intensity of monitoring for clinical worsening [27]. They discouraged the use of chest CT for diagnostic purpose of COVID-19; however, situations are different in a resource-constrained environment, in which availability of RT-PCR is limited or at emergency room, where patients are critical condition necessitating prompt triage or unconscious and unable to speak their symptoms or exposure history [27].

## Role of chest radiograph

Chest X-ray used to be deemed less useful than chest CT because of their lower sensitivity in the diagnosis of subtle parenchymal abnormalities and limited ability to help differentiate parenchymal patterns [28]. Chest CT is gold standard imaging technique for thoracic evaluation of COVID-19, but is not always available, for example, for unstable patients in the intensive care unit (ICU) with hypoxemia and hemodynamic failure [29]. For these patients, bedside chest X-ray is still the standard of care. Other advantages of chest X-ray include its ready and wide availability, making it possible to use in almost all clinical settings [28]. Chest X-ray is less-resource intensive, is achieved with lower radiation doses, is easier to repeat, and can be performed with portable equipment at the point of care, minimizing the risk of cross-infection related to patient transport [30]. Some early studies argued against the use of chest X-ray as the first-line imaging modality because of its low sensitivity in detecting alterations [31, 32]. In contrast, the later statements of several radiological societies have encouraged its use in combination with RT-PCR instead of CT [27, 33].

The sensitivity of chest X-ray depends mainly on two factors, i.e., symptom severity and disease stage [33, 34]. In relation to the former, Kuo et al. conducted a research to evaluate the screening value of chest X-ray with 1964 patients with COVID-19 who were asymptomatic or had mild symptoms as defined by the consensus statement of the Fleischner Society [35]. They demonstrated that only 39 patients (2.0%) showed abnormal findings on chest X-ray and full recovered after supplemental oxygenation and inpatient treatment [35]. The results of this study validated the Fleischner Society's proposal for the first clinical scenario, i.e., chest imaging is not recommended for asymptomatic or mildly symptomatic patients. Although the amount of research focused on the other two clinical scenarios, i.e., mild to severe patients with abundant or limited-resources, some research may provide hints to them [4, 33, 36, 36]. One is an early study by Chen et al., in which their first 99 cases of COVID-19 in Wuhan, China, were described [4]. The study cohort seemingly comprised moderate to severe hospitalized patients, 33% of whom had organ dysfunction, and 100% showing chest radiograph abnormality on admission [4]. Several other studies investigated a less severe spectrum of patients [36, 37]. Wong et al. investigated a cohort of 64 patients (86% symptomatic and 14% asymptomatic), 69% of whom showed chest radiograph abnormalities on admission [36]. Toussie et al. investigated a cohort of 338 patients (43% inpatients and 57% outpatients), 50% of whom showed abnormalities on the first chest radiograph at a mean of 4 days after onset [37]. The sensitivity of chest X-ray as a function of disease course was investigated by Vancheri et al., who recruited 240 mildly symptomatic patients with COVID-19 [33]. They showed that the sensitivity of chest X-ray was 63.3% on day 0–2, 72% on day 3–5, 81.2% on day 6–9, and 83.9% on day > 9 [33].

In summary, for the general population, chest X-ray is not recommended as the first-line imaging modality for early disease or asymptomatic or mildly symptomatic patients because of limited sensitivity compared to CT [35, 38]. In contrast, for those with progressed or moderate to severe disease, chest X-ray may be an effective alternative for assessing disease progression; the need for chest CT may be negated with positive chest X-ray findings [39]. For patients sensitive to radiation exposure, i.e., pregnant women or pediatric patients, or unstable patients unable to be transported to the CT room, chest X-ray is a useful alternative method of chest CT [29, 40].

## Role of ultrasound

With experience lung ultrasonography can be as useful as chest CT and superior to standard chest X-ray for evaluation of pneumonia and/or adult respiratory distress syndrome [40]. It has moreover the added benefits of ease of use, repeatability, no radiation exposure, and being cheap [40]. Point-of-care ultrasound using a hand-held mobile device enables assessments in various settings not only in emergency department and intensive care unit, but rural healthcare facilities, nursing homes, and aeromedical transport as well [41]. The appropriate use of ultrasonography could decrease chest X-ray and CT use in patients in the ICU [40].

Ultrasonography artefacts arising from the chest wall and pleural surfaces can provide valuable information about diverse lung pathologies either correlating or not correlating with the existing lung pathology of COVID-19 [40]. The normal lung back reflects ultrasound waves providing a transverse parallel hyperechoic lines called A-line [38]. With disease progression, new signs including pleural line (A-line) thickening and irregularities and B-line artifact, vertical hyperechoic lines starting from the pleura and continuing to the bottom of the image, may be noted [38, 42]. The presence of B-line will vary among focal, multifocal, and confluent patterns of involvement [40]. As B-lines reflect interstitial thickening and inflammation, the number increases with disease severity [42]. Consolidation and increased echogenicity of lung parenchyma with air-filled bronchi may also become apparent and increase in frequency and size [42]. The extent of consolidation may also vary becoming more prominent with the assumption of diverse patterns, from multifocal, small, subpleural consolidations to non-translobar and translobar involvement, and in some cases accompanied by air bronchograms [40]. The most specific finding of pneumonia is “Shred Sign”, which reflects an irregular shredded appearance at the interface between aerated normal and consolidated lung [38]. Pleural effusions are uncommon, with generally only those patients who are more critically ill showing them [40]. The presence of A-lines through the recovery phase is considered an indirect sign of recovery [40].

A meta-analysis by Barssoum et al. showed a sensitivity of lung ultrasound of 68–93.3% and of NPV of 52–94.1%, highlighting the value of lung ultrasound as a screening test to rule out COVID-19 pneumonia [43–47]. In contrast, the available data regarding specificity and PPV are conflicting with one study showing 92.9% and 84.6% for sensitivity and PPV, respectively, and another lower values of 21.3% and 19.2%, respectively [44, 47]. Lu et al. investigated the diagnostic performance of lung ultrasound with chest CT as the reference demonstrating high sensitivity and specificity in mild, moderate, and severe lung lesions with 68.8%, 77.8%, and 100.0% and 85.7%, 76.2%, and 92.9%, respectively [44].

## Standard reporting system of COVID-19 pneumonia

One unavoidable issue of the early radiological studies of COVID-19 was the subjectivity of image interpretation because of the insufficient evidence available [19–22]. In addition, early studies evaluated imaging findings in a binary fashion, COVID-19 positive or not, without considering the specificity of the CT findings [15–17]. However, this is in contrast to conventional clinical practice, where imaging findings are reported together with a differential diagnosis with different probabilities. Although COVID-19 presents a wide spectrum of imaging findings from typical (and highly specific) findings to atypical (and low specific) findings that overlap with those of other conditions, for the pursuit of high sensitivity, the discussion of specificity was not sufficient.

Accumulation of evidence has enabled radiologists to classify imaging findings by different specificities and establish standard reporting and CT categorization systems based on the probability of COVID-19 [48]. To date, four major systems have been proposed as summarized in Table 1 [49]. The use of categorical CT grading systems facilitates objective and uniform interpretation of CT and smooth communication with professionals from different fields and with different experiences [48, 50]. The first to be published was by the British Society of Thoracic Imaging (BSTI) which issued the Guidance for the Reporting Radiologist as a diagnostic framework of COVID-19 from chest CT and X-ray (BSTI classification) on March 16, 2020 [51]. BSTI classification comprises four categories, i.e., “CLASSIC COVID-19” (100% confidence for COVID), “PROBABLE COVID-19” (71–99% confidence for COVID), “INDETERMINATE” (< 70% confidence for COVID), “NON-COVID” (70% confidence for alternative) [51].

**Table 1:** Comparison of the chest CT categorization systems of COVID-19 [49]

Level of suspicion	CO-RADS Category	COVID-RADS category	The RSNA expert consensus statement category	The BSTI guideline statement category
Not interpretable	CO-RADS 0 (Scan technically insufficient for assigning a score)	Not defined	Not defined	Not defined
Very low	CO-RADS 1 (Normal or noninfectious)	COVID-RADS 0 (Normal)	Negative for pneumonia (No features of pneumonia)	NON-COVID (70% confidence for alternative)
		COVID-RADS 1 (Atypical findings; noninfectious etiology or infectious etiology but inconsistent with COVID-19)		
Low	CO-RADS 2 (Typical for other infection but not COVID-19)		Atypical appearance (Uncommonly or not reported features of COVID-19 pneumonia)	
Equivocal/unsure	CO-RADS 3 (Features compatible with COVID-19 but also other diseases)	COVID-RADS 2A (Fairly typical findings) COVID-RADS 2B (Combination of atypical findings with typical/fairly typical findings)	Indeterminate appearance (Nonspecific imaging features of COVID-19 pneumonia)	INDETERMINATE (< 70% confidence for COVID)
High	CO-RADS 4 (Suspicious for COVID-19)			PROBABLE COVID-19 (71–99% confidence for COVID)
Very high	CO-RADS 5 (Typical for COVID-19)	COVID-RADS 3 (Typical findings)	Typical appearance (commonly reported imaging features of greater specificity for COVID-19)	CLASSIC COVID-19 (100% confidence for COVID)
Proven	CO-RADS 6 (RT-PCR positive for SARS-CoV-2)	Not defined	Not defined	Not defined

*RSNA* Radiological Society of North America, *CO-RADS* the COVID-19 reporting and data system, *COVID-RADS* the COVID-19 imaging reporting and data system, *BSTI* British Society of Thoracic Imaging, *RT-PCR* Real-time reverse transcription polymerase chain reaction

**Fig. 1:**
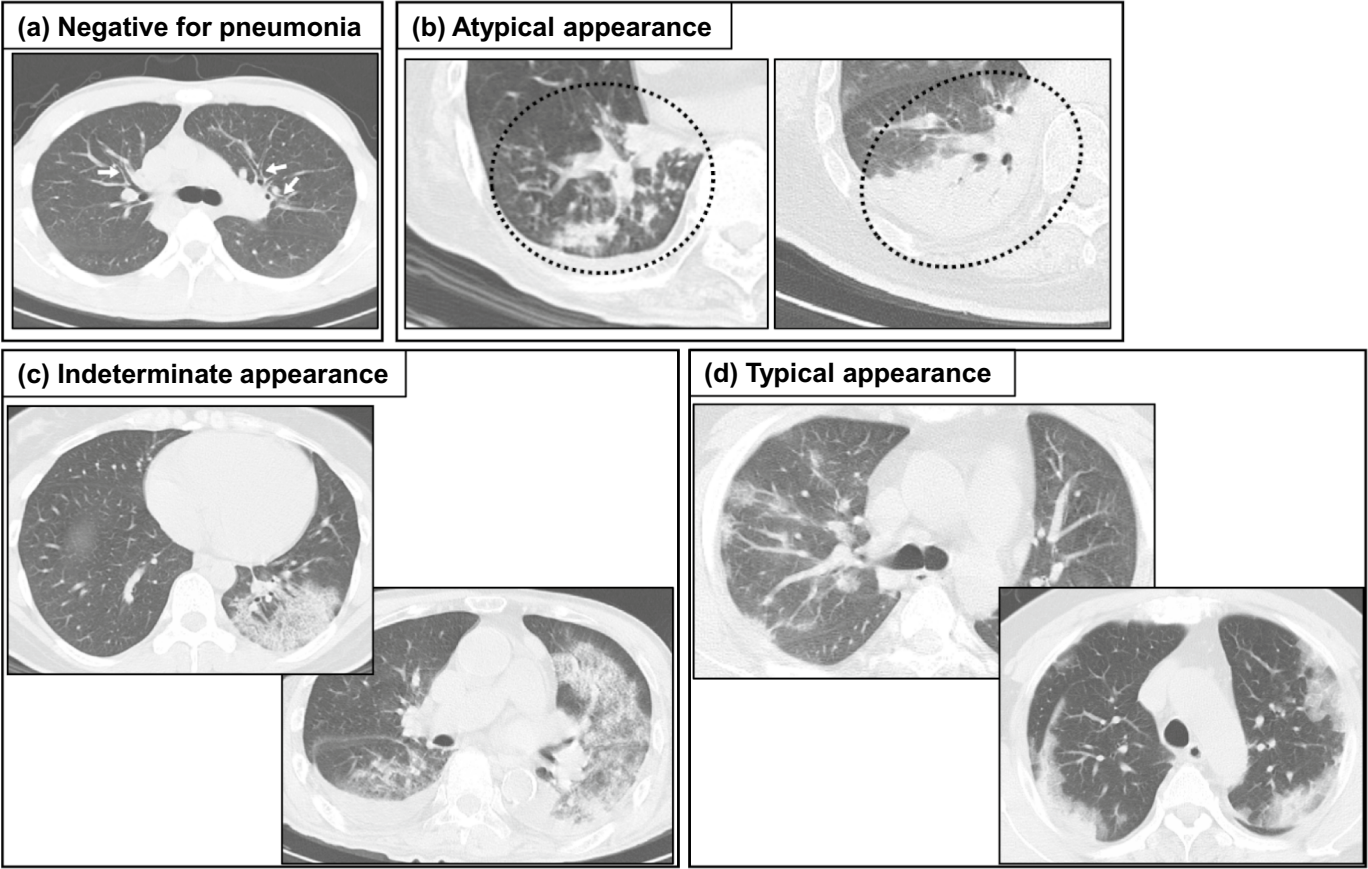
Example of chest CT patterns of RSNA classification. Axial CT images are categorized as (**a**) “Negative for pneumonia” meaning no features of pneumonia, (**b**) “Atypical appearance”, meaning typical for other infection but not COVID-19, e.g., bronchial pneumonia, lobar pneumonia, tuberculosis, or fungal infection, (**c**) “Indeterminate appearance”, meaning the presence of feature suspicious for COVID-19 but with overlaps with other diseases, drug-induced pneumonia, collagen disease-related lung diseases, or alveolar pulmonary edema, and (**d**) “Typical appearance”, meaning the typical pattern of COVID-19 pneumonia [49]

On March 21, 2020, the Radiological Society of North America (RSNA) advocated an expert consensus statement on reporting a standard nomenclature and imaging classification for COVID-19 pneumonia (RSNA classification) made up of four categories (“typical appearance”, “indeterminate appearance”, “atypical appearance”, and “negative for pneumonia”) [52] (Fig. 1) [49]. “Typical appearance” is defined as the presence of three imaging features: (1) peripheral and bilateral GGO, (2) multifocal round-shaped GGO, (3) organizing pneumonia pattern including reversed halo sign. GGO is often associated with intralobular lines showing a “crazy-paving pattern” [52]. If GGO exists but their distribution or shape is not typical, it is assigned to the “indeterminate appearance” category, which is defined as the two types of imaging features: (1) multifocal and diffuse but nonperihilar or unilateral nonrounded GGO, (2) few very small GGO with a nonrounded and nonperipheral distribution [52]. If GGO is not present and other causes of pneumonia or interstitial pulmonary edema are surmised, it is categorized as “atypical appearance” [52]. If no features of pneumonia exist, it is categorized as “negative for pneumonia” [52].

**Fig. 2:**
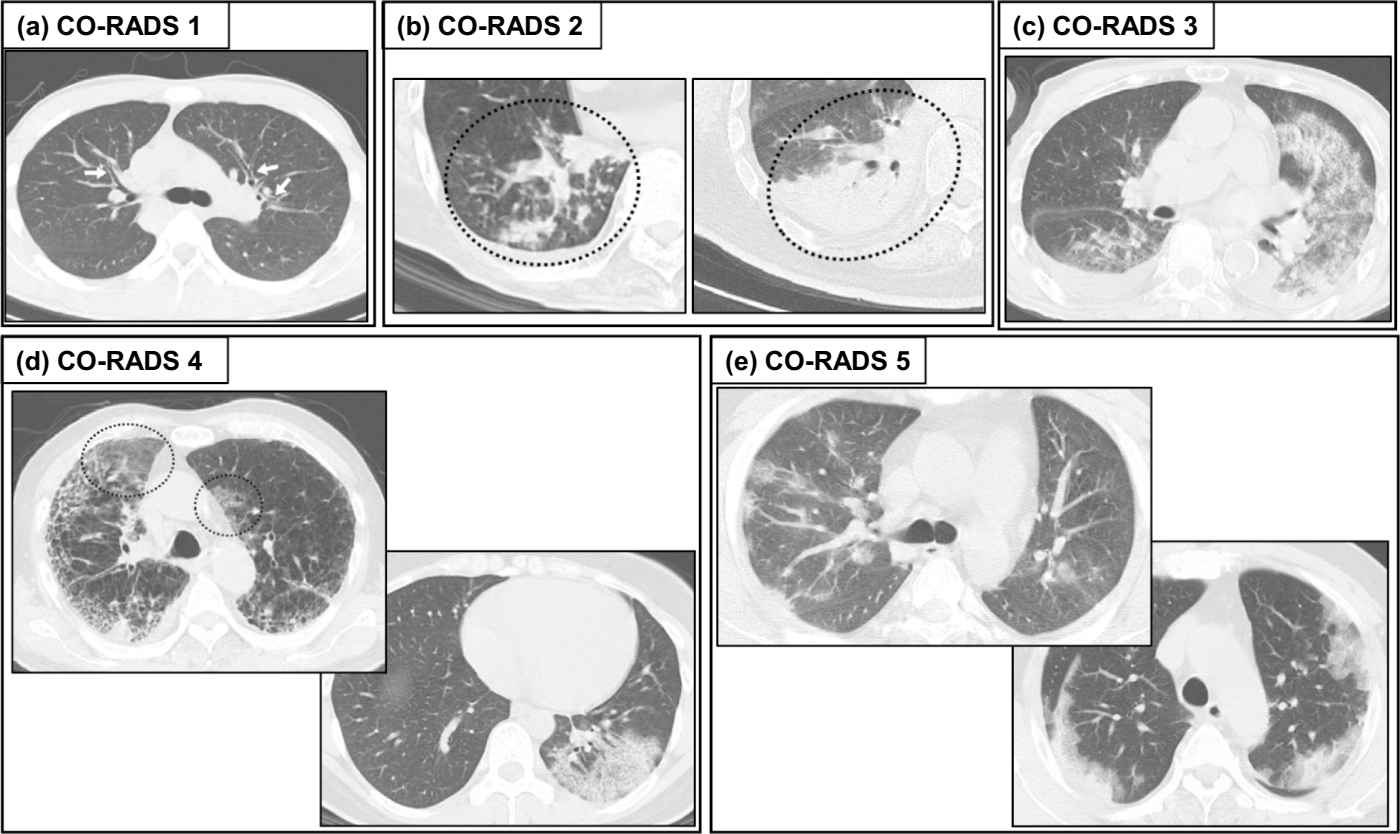
Example of chest CT patterns of CO-RADS. Axial CT images are categorized as (**a**) CO-RADS 1, with no features of pneumonia, (**b**) CO-RADS 2, with features typical for infection other than COVID-19, e.g., bronchial pneumonia, lobar pneumonia, tuberculosis, or fungal infection, (**c**) CO-RADS 3, with features compatible with COVID-19 but also other diseases, e.g., alveolar pulmonary edema, (**d**) CO-RADS 4, with features suspicious for COVID-19 but with overlap with other diseases, drug-induced pneumonia or collagen disease-related lung diseases, and (**e**) CO-RADS 5, with typical pattern of COVID-19 pneumonia [49]

On April 27, a working group of the Dutch Radiological Society proposed the COVID-19 reporting and data system (CO-RADS) to facilitate the advances in and worldwide dissemination of COVID-19 related information and tools [53]. As shown Table 1, CO-RADS 1 mostly corresponds to RSNA “negative for pneumonia”, CO-RADS 2 to RSNA “atypical appearance”, and CO-RADS 5 to RSNA “typical appearance” [53]. To put it simply, RSNA “indeterminate appearance” was divided into CO-RADS 3 and 4; isolated peripheral GGO or multifocal peripheral but unilateral GGO is categorized as CO-RADS 4 and other GGO as CO-RADS 3 [5] (Fig. 2) [49].

On April 28, researchers of the University of Southern California devised a different structured reporting system based on a review of 37 published papers on the chest CT findings of COVID-19 entitled the COVID-19 imaging reporting and data system (COVID-RADS) that divides the CT findings into five categories (COVID-RADS 0, 1, 2A, 2B, and 3) [54].

## Performance of BSTI classification

The diagnostic performance of BSTI classification of chest X-ray for symptomatic patients was evaluated in 4 studies from the UK [55–58]. The sensitivity and specificity greatly differed among different study populations probably because of different reference standards or imaging qualities or variabilities of interpretation [55–58]. A case–control study recruiting 50 patients each with or without COVID-19 by Hare et al. reported that “CLASSIC or PROBABLE COVID-19” showed a sensitivity, specificity, positive predictive value (PPV), and negative predictive value (NPV) of 44%, 100%, 46.8%, and 94.3%, respectively with reference to RT-PCR [55]. Dichotomization of “CLASSIC or PROBABLE COVID-19 or INDETERMINATE” and “NON-COVID” increased sensitivity to 70% at a specificity of 76% [55]. A cohort study by Kemp et al. reported that “CLASSIC COVID-19” showed a sensitivity, specificity, PPV, and NPV of 57.6%, 75.4%, 68.0%, and 66.2%, respectively [56]. A cohort study by Tsakok et al. reported that “CLASSIC or PROBABLE COVID-19” showed a sensitivity, specificity, PPV, and NPV of 61%, 76%, 63%, and 75%, respectively, with reference to RT-PCR [57]. A propensity-matched cohort study by Borakati et al. reported that “CLASSIC COVID-19” showed a sensitivity, specificity, PPV, and NPV of 56%, 60%, 71%, and 43%, respectively, with reference to RT-PCR [58].

The diagnostic performance of BSTI classification of chest CT was investigated in 2 studies from the UK and Japan [49, 55]. A cohort study by Borakati et al. reported that “CLASSIC COVID-19” showed a sensitivity, specificity, PPV, and NPV of 85%, 50%, 75%, and 66%, respectively, with reference to RT-PCR [55]. A case–control study by Inui et al. reported that “CLASSIC COVID-19” showed a sensitivity, specificity, PPV, and NPV of 64.5%, 92.0%, 91.5%, and 72.6%, respectively, with reference to RT-PCR [49]. Thresholding at “CLASSIC or PROBABLE COVID-19” increased sensitivities to 71.3% and “CLASSIC or PROBABLE COVID-19 or INDETERMINATE” to 91.3% [49].

Interobserver agreements for BSTI classification were studied by one study each for chest X-ray and chest CT [49, 55]. For chest X-ray classification, a cohort study by Hare et al. reported a moderate overall agreement with Fleiss’ kappa of 0.50 among 6 radiologists [55]. For chest CT classification, a cross-sectional study by Inui et al. reported moderate to good agreements with 0.54, 0.61, and 0.54 with Fleiss’ kappa, Cohen’s kappa, and Light’s kappa, respectively [49].

## Performance of RSNA classification

Diagnostic performance of the RSNA classification was evaluated for symptomatic patients with reference to RT-PCR as summarized in Table 2 [49, 59–66]. A retrospective single-center cohort study from Italy that included 460 symptomatic patients with suspected COVID-19 from February 27 to March 27, 2020, showed that the sensitivity and specificity of “typical appearance” were 71.6% and 91.6% [61]. When “typical appearance” and “indeterminate appearance” were grouped together, the sensitivity increased to 88.6% [61]. They also revealed that PPV of “typical appearance” was 40.0% at a prevalence of 16.7% and increased to 87.8% at 46.4% [61]. Similar trends were observed in reports from other countries, i.e., Brazil [60, 63, 66, 67], Japan [49], and USA [64] and other institutions in Italy [61, 62, 65]. Regarding the diagnostic performance against other viral pneumonias, a retrospective cohort study from Brazil reported similar results with a sensitivity and specificity of 73.6% and 97.7%, respectively, for “typical appearance” [65]. They also evaluated the CT classification in relation to the duration of symptoms, which showed that “indeterminate appearance” and “negative for pneumonia” were significantly more frequent in patients with symptom duration of 0–5 days than > 5 days, suggesting milder CT patterns in the early stage of infection [66]. A retrospective cohort study from Italy that compared the diagnostic performance in relation to age reported that PPV, NPV, and accuracy were not statistically different among the different age groups in their study comprising patients > 60, ≥ 50 and < 60, or < 50 [62].

**Table 2:** Performance of RSNA classification

First author	Country	Inclusion period [in 2020]	Number of patient	RT-PCR positivity (%)	Typical appearance				Typical or indeterminate appearance			
					Sen	Spe	PPV	NPV	Sen (%)	Spe (%)	PPV (%)	NPV (%)
Barbosa [60]	Brazil	Feb to Mar	91	28	64%	85%	61%	86%	92	62	48	95
Cicaresse [61]	Italy	2/27–3/27	460	46	72%	92%	88%	79%	89	67	69	87
Falaschi [62]	Italy	3/3–4/9	773	60	—	—	—	—	91	79	86	85
Santos [63]	Brazil	3/13–3/23	71pt/75CT	45	83%	97%	95%	87%	92	79	78	92
Som [64]	USA	1/15–3/30	89	40	86%	80%	74%	89%	98	55	60	98
Colombi [65]	Italy	3/30–4/13	239	45	—	—	—	—	85	69	69	85
Inui [49]	Japan	1/30–6/30	100	50	74%	83%	81%	76%	92	41	61	84
De Saliva Teles [66]	Brazil	3/15–3/24	175	50	74%	98%	97%	79%	83	88	87	84

*RSNA* Radiological Society of North America, *RT-PCR* real-time reverse transcription polymerase chain reaction, *Sen* sensitivity, *Spe* specificity, *PPV* positive predictive value, *NPV* negative predictive value, *pt* patients, *CT* computed tomography

Interobserver agreements could not be directly compared because of the difference in methodology, indicators, or observer experiences among studies [49, 64, 68]. A cohort study by de Jaegere et al. reported moderate to good interobserver agreement with a weighted kappa of 0.57–0.66 between two chest radiologists and a radiology resident [68]. A cross-sectional study by Inui et al. included 8 radiologists with different length of experience (4 less than 10 years and 4 more than 10 years) and held an experimental training session to familiarize all of the observers with the categorization systems beforehand [49]. They showed moderate to good interobserver agreement across observers with an average Cohen’s kappa of 0.63 and Light’s kappa of 0.55 [49]. Another cohort study by Som and Lang et al. that included 6 radiologists with experience durations ranging 1–15 years reported moderate to high interobserver agreements among attending radiologists with kappa values of 0.43–0.86 and moderate agreements among trainee radiologists with kappa values of 0.62 and 0.77 [64].

Factors underlying interobserver disagreements were investigated in these studies. One study revealed that the presence of co-existing lung diseases affected interobserver agreements because COVID-19 pneumonia often mimics acute aggravation of interstitial pneumonia (IP) or emphysema when superimposed on them [49]. Another study employed a self-reported certainty scoring method, in which each observer noted his/her confidence of each categorization [64]. They found that disagreements were associated with two or more dominant findings suggestive of multiple diseases, minimal disease, and an ambiguous distribution or morphology of findings [64]. They also identified atelectasis, nodular morphology, presence of pre-existing disease, and a peribronchial pattern suggestive of organizing pneumonia as sources of uncertainty [6].

## Performance of CO-RADS

Diagnostic performance of CO-RADS was evaluated in both symptomatic and asymptomatic patients with reference to RT-PCR as summarized in Table 3 [49, 59, 68–78]. A retrospective single-center cohort study from the Netherlands that included 1070 symptomatic patients with suspected COVID-19 from March 22 to April 7, 2020, showed that the sensitivity and specificity of CO-RADS 5 were 71% and 88%, respectively. [77]. They also showed the sensitivity increased to 86% at the threshold of ≥ CO-RADS 4 and 92% at ≥ CO-RADS 3. The PPV and NPV for CO-RADS 5 were reported to be 86% and 75% at the incidence of 50%, respectively [77]. Similar trends were observed in the subsequent reports from other countries including Belgium [70], France [75], Germany [78], Italy [73, 74], and Japan [49, 71] and other institutions from the Netherlands [68, 69, 72, 76, 77].

**Table 3:** Performance of CO-RADS

First author [reference]	Country	Inclusion period [in 2020]	No. of patient	RT-PCR positivity (%)	CO-RADS 5				≥ CO-RADS 4				≥ CO-RADS 3			
					Sen	Spe	PPV	NPV	Sen (%)	Spe (%)	PPV (%)	NPV (%)	Sen	Spe	PPV	NPV
de Jaegere [68]	Netherlands	3/12–3/23	96	47	62%	98%	96%	75%	84	92	91	87	–	–	–	–
Hermans [69]	Netherlands	3/27–4/20	319	42	75%	94%	90%	84%	90	88	85	93	93%	66%	66%	93%
De Smet [70]	Belgium	3/19–4/20	859	42	78%	93%	89%	86%	85	85	80	89	89%	85%	69%	90%
Fujioka [71]	Japan	Jan-Jun	154	49	53%	94%	90%	67%	68	88	85	74	–	–	–	–
Korevaar [72]	Netherlands	3/16–4/16	239	47	–	–	—	–	93	70	73	92	97%	51%	64%	96%
Bellini [73]	Italy	3/9–5/3	572	23	–	–	–	–	61	81	50	87	–	–	–	–
Tucato [74]	Italy	Unspecified	120	43	91%	91%	92%	90%	75	75	77	72	74%	76%	77%	72%
Nivet [75]	France	3/13–4/14	513	48	79%	97%	96%	84%	92	84	84	92	96%	43%	60%	92%
Inui [49]	Japan	1/30–6/30	100	50	65%	89%	86%	72%	86	68	73	83	91%	54%	66%	86%
Lieveld [76]	Neherlands	3/19–3/28	741	50	–	–	–	–	89	87	76	95	95%	54%	49%	96%
Schalekamp [77]	Netherlands	3/20–4/3	1070	50	71%	88%	86%	75%	86	81	82	85	92%	66%	73%	89%
Gross [78]	Germany	3/22–4/7	96	20	80%	99%	95%	95%	90	91	71	97	90%	63%	38%	96%

*CO-RADS* the COVID-19 reporting and data system, *RT-PCR* real-time reverse transcription polymerase chain reaction; Sen, sensitivity, *Spe* specificity, *PPV* positive predictive value, *NPV* negative predictive value

Regarding the diagnostic performance for asymptomatic patients, a retrospective cohort study from a pandemic area in Belgium included 859 asymptomatic and 1138 symptomatic patients with suspected COVID-19 [70]. The sensitivity was lower but the specificity was higher in asymptomatic than symptomatic patients at the thresholds of CO-RADS 3 to CO-RADS 5 (e.g., sensitivity and specificity, 78% and 93% in symptomatic patients for CO-RADS 5 vs 18% and 98% in asymptomatic one) [70]. For asymptomatic patients with an incidence of 5.8%, judgments of COVID-19 positive at the thresholds of ≥ CO-RADS 3 increased the posttest probability to 18–32% with specificity of 89–98% [70]. However, judgments of COVID-19 negative only slightly decreased the posttest probability to 3.3–4.4% with sensitivity of 18–45% [70]. Judging from these results, chest CT patterns of ≥ CO-RADS 3 in asymptomatic patients raise strong suspicion of COVID-19, while those < CO-RADS 3 do not rule it out, arguing against the use of chest CT as a screening test for asymptomatic individuals. On the other hand, for symptomatic patients with an incidence of 42%, only CO-RADS 5 increased the posttest probability to 89% [70]. Therefore, from the standpoint of infection control, CO-RADS 5 may be used as a triage tool to quarantine symptomatic individuals in settings with bottlenecks in RT-PCR tests.

In relation to the duration of symptoms, CO-RADS achieved the highest diagnostic performance in patients with a symptom duration of 2–7 days, followed by those with symptom durations of more than 7 days and less than 2 days [77]. Abdel-Tawab et al. investigated 359 patients with COVID-19 and showed that the chest CT positivity as defined by ≥ CO-RADS 3 was only 9.4% for asymptomatic/mild symptomatic group, which increased to 94.7% and 97.8% for moderately and severely symptomatic groups, respectively [48]. They also showed that chest CT positivity is roughly parallel to age groups as defined by < 15 years, 15–50 years, or > 50 years [48]. A head-to-head comparison study from Japan showed that the diagnostic performance of CO-RADS exceeded that of RSNA classification and COVID-RADS (area under the receiver operating characteristic curve: AUCs; 0.84.xx for CO-RADS versus 0.81 for RSNA classification and 0.80 for COVID-RADS) [49].

Interobserver agreements of CO-RADS also vary in methods among studies. A cohort study by de Bellini et al. recruited 9 radiologists and reported substantially varied results between different levels of experience with a Fleiss’ kappa of 0.38–0.66 [73]. A cohort study by de Jaegere et al. reported good interobserver agreement with a weighted kappa of 0.65–0.77 between two chest radiologists and a radiology resident [68]. A cross-sectional study by Inui et al. reported good agreement with Fleiss’ kappa of 0.62 among 8 observers [49]. Some studies used an interclass coefficient (ICC) as an indicator [48, 71]. However, ICC is the best option for use with continuous variables or categorical variables with the ordinal scale of the same distance between categories (e.g., score 1–5) [79]. Instead, the CT categorization systems are nominal scales that numbered (e.g., CO-RADS 0–6) groups of CT patterns and therefore, the use of ICC is not recommended.

**Fig. 3:**
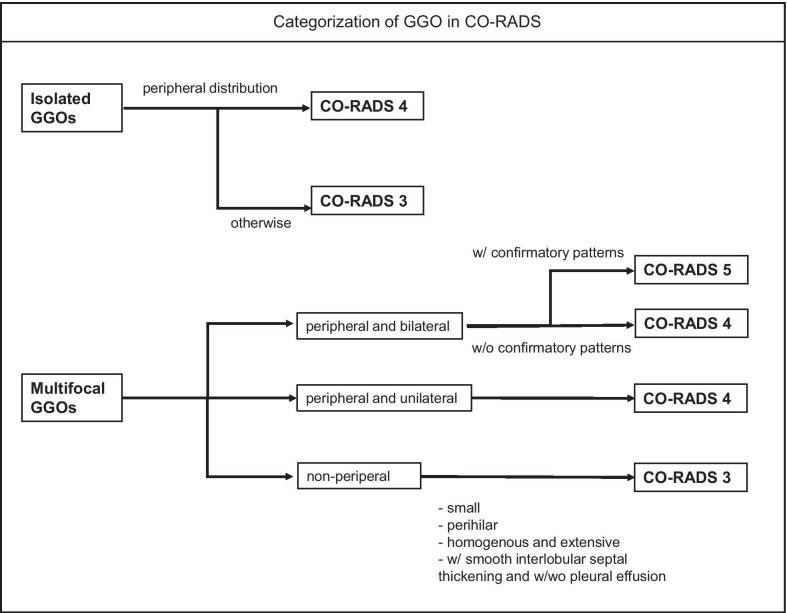
Flow chart illustrating the categorization of ground-glass opacity in CO-RADS

CO-RADS is a detailed categorization system but has the one potential limitation of complexity of its GGO categorization [49]. To resolve this issue, Inui et al. proposed the use of sample CT patterns that may facilitate the understanding and categorization work as summarized in Fig. 3 [49]. With this workflow, first GGOs incompatible with CO-RADS 1 or 2 are classified into isolated GGOs regardless of size [49]. For an isolated GGO, those with peripheral distribution fall into CO-RADS 4 and not CO-RADS 3 [49]. For multifocal GGOs, lesions are further classified into peripheral or non-peripheral distribution [49]. Multifocal, peripheral and bilateral GGOs fall into CO-RADS 5 if the confirmatory patterns are present and CO-RADS 4 if not [49]. Multifocal and peripheral but unilateral GGOs are categorized as CO-RADS 4 regardless of the presence of the confirmatory pattern [49]. Then, non-peripheral lesions are categorized as CO-RADS 3 regardless of laterality. Such lesions include small, perihilar, and homogeneous extensive ones [49].

Another possible solution to avoid the complexity of CO-RADS categorization is to an artificial intelligence (AI)-based algorithm created by the authors of CO-RADS original article [80]. This software is available for free, works in the local computer, and automatically perform CO-RADS categorization from DICOM files of chest CT [80]. The performance of AI-based system was externally validated in the original article with an AUC of 0.95 and 0.88 in internal and external cohorts, respectively [80].

## Performance of COVID-RADS

Only a single study evaluated the performance of COVID-RADS [49]. The sensitivity and specificity of COVID-RADS 3 were 65.5% and 90.0%, respectively [49]. Dichotomization of suspected CT with “COVID-RADS 2A or 3” or “COVID-RADS 2B or 3” and “COVID-RADS 0 or 1” increased the sensitivities to 85.5% and 91.0% at specificities of 68.3% and 53.8%, respectively [49]. Interobserver agreements of COVID-RADS were 0.63 and 0.55 as assessed by average Cohen’s kappa and Light’s kappa, respectively [49].

## Proportion of COVID-19 by category

The proportion of negative CT findings for pneumonia (CO-RADS 1, RSNA classification “negative for pneumonia”, COVID-RADS 1, or BSTI classification “NON-COVID”) was roughly 5–15% in symptomatic patients with COVID-19 [49]. This percentage corresponds to the previously reported incidence of symptomatic patients with COVID-19 but without CT abnormalities [22, 23]. Excluding categories of negative CT findings, the proportion of COVID-19 positivity for each category was roughly parallel to the level of suspicion of the category in each classification system [49]. In some studies, however, a reversed incidence was observed in the lowest two categories [49, 61, 63, 66, 68–70, 75]. For example, the proportion of CO-RADS 1 (negative CT findings) was higher  than CO-RADS 2 (suspected pneumonia caused by other pathogen), and a similar trend was seen in RSNA “negative for pneumonia” and RSNA “atypical appearance”, COVID-RADS 1 and 2, or BSTI “NON-COVID” and “INDETERMINATE” [49, 68–70, 75]. This may be easily understood because the incidence of CT negative patients in COVID-19 and pneumonia of other etiology may be changeable depending on the inclusion criteria among community-acquired infection populations. Specifically, CT positivity is roughly parallel to the severity of symptoms and age and dependent on disease stage [48, 77].

## Comparison of the proposed CT categorization systems

Each of the proposed standard reporting system has its own pros and cons. The RSNA and BSTI classifications are easy to interpret and categorize based on gestalt imaging interpretation of CT, as their categories are assigned according to specific patterns [49]. One disadvantage of the RSNA classification is that it does not address any co-existing lung diseases, thereby probably compromising the diagnostic performance, as discussed above [49, 64]. In contrast, CO-RADS is seemingly complex because its categorization of GGO is more detailed (3 categories in CO-RADS vs. 2 categories in the other) and employs more rigorous definitions [49]. However, this kind of detailed categorization enhances the overall diagnostic performance and enables more effective optimization of diagnostic thresholds depending on the pre-test probabilities of the corresponding area. In addition, broad evidence is available regarding its clinical application in various settings [49, 59, 68–78]. Meanwhile, complexity may be the only disadvantage, which may be resolved by use of the proposed flow chart (Fig. 3). COVID-RADS is also a complex system that assigns scores for each CT finding, while the overall score is defined by a combination of them [49]. One disadvantage of COVID-RADS is that it does not consider zonal distribution of lesions on the axial plane [49]. In addition, it categ orizes “multifocal GGO” as a “typical finding” but there may be overlap with other diseases including bronchial, viral, or fungal pneumonia, lymphoproliferative diseases, and early-stage interstitial pneumonia [49]. Moreover, typical patterns of COVID-19 may be downgraded from COVID-RADS 3 to 2B when they are accompanied with “fairly typical findings” including a small amount of pleural effusion, pulmonary, emphysema, or fibrosis, with many such COVID-19 cases encountered [49]. In summary, the authors conclude that CO-RADS is the most balanced, effectively optimized, and evidence-based method among the proposed reporting systems.

## Clinical course of COVID-19 pneumonia

Defining disease stage and predicting and monitoring disease progression are major roles of chest imaging. A typical pattern of disease progression of COVID-19 starts as subpleural non-segmented GGOs or small rounded GGOs in the middle layer of the lung parenchyma that distribute unilaterally or bilaterally, culminating in a crazy-paving pattern and subsequent consolidation [81, 82]. According to Pan et al., chest CT patterns can be divided into the following 4 stages: (1) early stage (day 0–4), when the main findings are GGOs occasionally accompanied by crazy-paving patterns that distribute unilaterally or bilaterally (chest CT findings may be negative at this stage), (2) progressive stage (day 5–8), when regions progressively congregate and extend bilaterally, with increased frequency and proportion of crazy-paving and consolidation, (3) peak stage (day 9–13), when regions slowly expand with an increasing proportion of consolidation and peaks out often accompanied with an organizing pneumonia pattern, i.e., volume reduction, perilobular distribution, and/or residual parenchymal band, and (4) absorption stage (days ≥ 14), when consolidations are gradually absorbed with GGOs being the most frequent findings [81]. The disease severity peaks at 6–11 days on chest CT and 10–12 days on chest X-ray after symptom onset [35, 81].

## Prognostic value of chest imaging findings

Some imaging findings were revealed to be useful to predict the clinical course or prognosis of COVID-19. Columbi et al. evaluated the percentage of well-aerated lung area visually or with a computer-aided method, in which normal opacity area less than 71–73% was associated with ICU admission or death with odds ratio (OR) of 5.4 [83]. They also showed that patients who were admitted to ICU or died had a higher percentage of ≥ 4 affected lobes [83]. Li et al. also investigated the relationship between prognosis and pneumonia extension as evaluated by the chest CT score, in which patients who died had higher CT scores on the initial examination in those over 60 years [84]. Lieveld et al. showed that the chest CT score was significantly positively associated with hospital and ICU admission, and in-hospital and 30-day mortality for all age groups in patients with COVID-19 and CT patterns ≥ CO-RADS 3 [76]. Abdel-Tawab et al. showed that all death cases occurred in patients with CO-RADS 5 and “typical appearance” of RSNA classification, suggesting possible prognostic value of the CT categorization systems [48].

Similar results were obtained from studies that investigated chest X-ray on admission. Toussie et al. scored chest X-ray on a 7-point scale for each lung in young and middle-aged patients [36]. They showed that a total score of ≥ 2 was associated with hospital admission (OR 6.2) and total score of ≥ 3 was an independent predictor of intubation (OR 4.7) in young and middle-aged patients. Schalekamp and Huisman et al. reported that distribution of lung disease on chest X-ray and chest X-ray severity score was associated with ICU admission and/or death [85].

One notable fact to keep in mind when interpreting evidence regarding the prognostic value of the initial chest CT or X-ray is that these studies were based on patients with moderate-to-severe lung involvement. The relationship between the degree of lung involvement in the initial chest imaging and prognosis is mostly applicable to asymptomatic or mildly symptomatic patients from the authors’ experience. However, in the authors’ experience, in some patients with mild symptoms but especially with risk factors, acute aggravation is often experienced after about 1 week of symptom onset, often resulting in death inside or outside the hospital. Therefore, caution is needed when applying the evidence to individual patients, and close monitoring is recommended for high-risk patients whose symptoms were even mild or the initial chest imaging findings were minimal.

## Long-term chest imaging findings

CT abnormalities may persist after recovery of COVID-19. Han and Fan et al. reported 6-month follow-up results of severe COVID-19 pneumonia [86]. Surprisingly, 40/114 (35%) of patients showed CT abnormalities suggestive of fibrotic change, which was associated with factors including age over 50, ARDS, and more prominent CT lung involvement [86]. This may be ascribed in part to the fact that the severity of the lung parenchyma injury may reflect the intensity of the repair process including hyaline membrane production and fibroblast accumulation, which later results in fibrotic changes [87–89]. They also showed that 27/104 (26%) of patients had abnormal diffusing capacity of the lung as assessed by carbon monoxide or DL_CO_, which was more frequent in those with fibrotic changes on CT [86]. Grist et al. investigated ^129^Xe MRI in dyspneic patients at 3-months after COVID-19 pneumonia [90]. They showed hyperpolarized Xe MRI abnormalities in comparison with healthy subjects in structurally and functional normal lung as assessed by CT and lung function test [90].

## Conclusion

Chest imaging is not recommended for routine screening of COVID-19 in a normal clinical situation. It still has an integral role to play, however, in its work up and staging, especially when assessing complications or disease progression. The most important role of radiologists in this context is to be able to identify those patients at greatest risk of imminent clinical decompensation by learning to stratify cases of COVID-19 on the basis of radiologic imaging in the most efficient and timely fashion possible. The present availability of multiple and more refined CT grading systems and classification is now making this task easier and thereby contributing to the recent improvements achieved in COVID-19 treatment and outcomes.

## Data Availability

Not applicable.

## Abbreviations

ACR: American College of Radiology
AI: Artificial intelligence
ARDS: Acute respiratory distress syndrome
ASER: American Society of Emergency Radiology
AUC: Area under the receiver operating characteristic curve
BSTI: British Society of Thoracic Imaging
CO-RADS: The COVID-19 reporting and data system
CoV: Coronavirus
COVID-19: Coronavirus disease 2019
COVID-RADS: The COVID-19 imaging reporting and data system
CT: Computed tomography
GGO: Ground-glass opacity
ICC: Interclass coefficient
ICU: Intensive care unit
IP: Interstitial pneumonia
NPV: Negative predictive value
OR: Odds ratio
PPV: Positive predictive value
RSNA: Radiological Society of North America
RT-PCR: Real-time reverse transcription polymerase chain reaction
STR: Society of Thoracic Radiology
WHO: World Health Organization
